# A clinical and *in vitro* assessment of outpatient parenteral benzylpenicillin and ceftriaxone combination therapy for enterococcal endovascular infections

**DOI:** 10.1093/jacamr/dlab128

**Published:** 2021-08-07

**Authors:** Paul R Ingram, Jacinta Ng, Claire Mathieson, Shakeel Mowlaboccus, Geoffrey Coombs, Edward Raby, John Dyer

**Affiliations:** 1 Department Infectious Diseases, Fiona Stanley Hospital, Perth, Australia; 2 School of Pathology and Laboratory Medicine, University of Western Australia, Perth, Australia; 3 Department of Microbiology, PathWest Laboratory Medicine, Murdoch, Western Australia, Australia; 4 College of Science, Health, Engineering and Education, Murdoch University, Perth, Australia

## Abstract

**Background:**

Amoxicillin plus ceftriaxone combination therapy is now standard of care for enterococcal endocarditis. Due to amoxicillin instability in infusion devices, benzylpenicillin plus ceftriaxone may be substituted to facilitate outpatient parenteral antimicrobial therapy (OPAT) delivery, despite lack of guideline endorsement.

**Objectives:**

To assess the clinical efficacy of benzylpenicillin plus ceftriaxone for the management of enterococcal endovascular infections, in addition to assessing this combination’s *in vitro* synergy.

**Patients and methods:**

Retrospective cohort study assessing unplanned readmissions, relapses and mortality for 20 patients with endovascular *Enterococcus faecalis* infections treated with benzylpenicillin plus ceftriaxone delivered via OPAT. For a subset of isolates, synergism for both amoxicillin and benzylpenicillin in combination with ceftriaxone was calculated using a chequerboard method.

**Results:**

Patients had endovascular infections of native cardiac valves (*n = *11), mechanical or bioprosthetic cardiac valves (*n = *7), pacemaker leads (*n = *1) or left ventricular assistant devices (*n = *1). The median duration of OPAT was 22 days, and the most frequent antimicrobial regimen was benzylpenicillin 14 g/day via continuous infusion and ceftriaxone 4 g once daily via short infusion. Rates of unplanned readmissions were high (30%), although rates of relapsed bacteraemia (5%) and 1 year mortality (15%) were comparable to the published literature. Benzylpenicillin less frequently displayed a synergistic interaction with ceftriaxone when compared with amoxicillin (3 versus 4 out of 6 isolates).

**Conclusions:**

Lower rates of synergistic antimicrobial interaction and a significant proportion of unplanned readmissions suggest clinicians should exercise caution when treating enterococcal endovascular infection utilizing a combination of benzylpenicillin and ceftriaxone via OPAT.

## Introduction

Enterococci are the third most frequent cause of infective endocarditis (IE).^1^ As guidelines recommend prolonged treatment,^2^ outpatient parenteral antimicrobial therapy (OPAT) is often utilized.^1^ Although *in vitro* growth is typically inhibited by penicillins with low MICs,^3^ enterococci are tolerant to the bactericidal effects of penicillins, often with minimum bactericidal concentrations (MBC) to MIC ratios more than 32.^3^ To overcome this, endocarditis is managed with high dose β-lactams, plus combination therapy selected on the basis of *in vitro* synergism.^2^ Either amoxicillin or benzylpenicillin in combination with an aminoglycoside is the traditional regimen for *Enterococcus faecalis* IE,^2^ however, frequent high-level aminoglycoside resistance or renal dysfunction have led guidelines to equally endorse amoxicillin in combination with ceftriaxone^2^—a regimen supported by *in vitro* data,^4^ animal model^5^ and human observational studies.^1^ Unlike benzylpenicillin, amoxicillin is too unstable for outpatient use.^6^ Thus, despite lack of guideline endorsement,^2^ benzylpenicillin and ceftriaxone are not uncommonly utilized for enterococcal IE management via OPAT.^6^^,^^7^

We aimed to assess the clinical efficacy of benzylpenicillin plus ceftriaxone therapy delivered via OPAT for the management of enterococcal endovascular infection, and assessed for *in vitro* synergy between this combination.

## Patients and methods

We performed a retrospective cohort study at a 783 bed Australian tertiary hospital with a multidisciplinary IE service as described previously.^8^ Patients were identified from a pre-existing, OPAT database, then restricted to those with endovascular infection caused by enterococci that were treated with benzylpenicillin plus ceftriaxone. For patients with IE, this was limited to those with definite IE as per the modified Duke criteria.^9^ Patient selection for OPAT was at the discretion of an infectious diseases (ID) physician. Community nurses administered antimicrobials via a peripherally inserted central catheter by slow injection or continuous infusion using elastomeric devices. All patients were managed by an ID physician, including weekly laboratory monitoring, clinical review and discussion at a multidisciplinary team meeting.

Patient, infection- and treatment-related information was extracted from medical records, using previously published definitions for immunosuppression^8^ and location of acquisition.^8^ Indications for surgical management of IE were assessed according to European guidelines.^2^ Outcomes assessed were unplanned readmissions, adverse events, relapsed bacteraemia and mortality.

Prior to initiation of benzylpenicillin therapy, all isolates were demonstrated to be penicillin susceptible via Vitek2 (bioMérieux, MO, USA).^10^ For six randomly selected isolates, MICs for amoxicillin, benzylpenicillin and ceftriaxone were determined by broth microdilution according to CLSI guidelines,^11^ and synergy testing was performed via the chequerboard method.^12^ Using commercially supplied powder (Sigma–Aldrich, USA) and cation-adjusted Muller-Hinton broth (Becton-Dickinson, USA), solutions of amoxicillin, benzylpenicillin and ceftriaxone were prepared, and then serially diluted. One hundred μL of amoxicillin, benzylpenicillin and ceftriaxone solution at concentration ranges of 0.008–8.0 mg/L, 0.008–8.0 mg/L and 0.5–32 mg/L, respectively, were prepared in a 96-well tray. Twenty μL of a 5 × 10^5^ cfu/mL solution of each isolate was inoculated into each well and the tray incubated at 35°C for 16–20 h. Using the MIC of either amoxicillin or benzylpenicillin alone, ceftriaxone alone and either amoxicillin or benzylpenicillin in the presence of 4 mg/L of ceftriaxone, the fractional inhibitory concentration index (FICI) was calculated.^12^*E. faecalis* ATCC 29212 and *Escherichia coli* ATCC 25922 were used as quality control strains and all experiments were performed in duplicate.

Multilocus sequence types were determined by whole genome sequencing. DNA was extracted using the DNeasy^®^ Blood and Tissue kit (Qiagen, 69506) according to the manufacturer’s instructions. DNA was quantified using the Qubit™ 3.0 Fluorometer (Thermofisher). DNA libraries were prepared using the Nextera^®^ XT DNA Library Prep kit (Illumina, United States) as per the manufacturer’s protocol. Libraries were sequenced on the Illumina NextSeq™ 500 platform using 150 bp chemistry. Raw reads were assembled using SPAdes v3.10.1,^13^ and sequence types were assigned using the *E. faecalis* MLST scheme^14^ on the PubMLST website (https://pubmlst.org).

The study was approved by the South Metropolitan Health Service Human Research Ethics Committee (RGS-1075).

## Results

Between 2015 and 2020 twenty patients were treated with benzylpenicillin plus ceftriaxone via OPAT for enterococcal endovascular infections (Table 1). The median duration of inpatient antimicrobial therapy prior to OPAT was 17 days and consisted of amoxicillin plus either ceftriaxone (*n = *13, 65%), or gentamicin (*n = *6, 30%) or benzylpenicillin plus gentamicin (*n = *1, 5%). During OPAT benzylpenicillin was always administered by continuous infusion, either at 14 g (*n = *15, 75%), 10.8 g (*n = *4, 20%) or 8.8 g (*n = *1, 5%) per day. All patients received 4 g/day of ceftriaxone, by once daily injection (*n = *11, 55%), continuous infusion (*n = *8, 40%) or 2 g twice daily (*n = *1, 5%). The median duration of antibiotic therapy via OPAT was 22 days, during which three (15%) patients experienced antimicrobial adverse effects. Unplanned readmission from OPAT occurred in six patients (30%), due to either congestive cardiac failure (one case of acute mitral valve perforation/regurgitation, one case of progressing mitral valve regurgitation), fever for investigation (both attributed to persisting infection, one managed successfully with no change to therapy and the other managed with palliative withdrawal of antimicrobials), arrhythmia (new onset atrial fibrillation) or antimicrobial adverse effect (drug rash with eosinophilia and systemic symptoms). Longer term follow-up revealed one patient (5%) experienced relapsed bacteraemia within 6 months of diagnosis, and 1 year mortality was 15%.

**Table 1.** Infection-related characteristics and outcomes for patients receiving benzylpenicillin in combination with ceftriaxone for enterococcal endovascular infections (*n = *20)

Characteristic	Value
Age, years, median (IQR)	69 (60–79)
Male, *n* (%)	14 (70%)
Aboriginal or Torres Strait Islander, *n* (%)	1 (5%)
Charlson co-morbidity index, median (IQR)	2.5 (0.5–3)
Baseline creatinine, μmol/L, median (IQR)	90 (66–145)
Immunosuppression, *n* (%)	4 (20%)
Risk factors for infective endocarditis, *n* (%)	
Prior valve surgery	10 (50%)
Prior infective endocarditis	3 (15%)
Intravenous drug use (past or present)	4 (20%)
Congenital heart disease	1 (5%)
Rheumatic heart disease	0 (0%)
Location of acquisition, *n* (%)	
Community onset, non-healthcare associated	11 (55%)
Community onset, healthcare associated	9 (45%)
Nosocomial	0 (0%)
Valvular involvement (can be >1), *n* (%)	
Aortic	13 (65%)
Mitral	5 (25%)
Tricuspid	1 (5%)
Pacemaker lead/LVAD	2 (10%)
Prosthesis involvement, *n* (%)	
Mechanical cardiac valve	4 (20%)
Bioprosthetic cardiac valve	3 (15%)
Pacemaker lead	1 (5%)
LVAD	1 (5%)
Inpatient length of stay, days, median (IQR)	17 (12–22)
Surgery management of infective endocarditis indicated, *n* (%)	12 (60%)
Surgery performed	7
OPAT antibiotic duration, days, median (IQR)	22 (8–34)
Catheter-related adverse event during OPAT, *n* (%)	0 (0%)
Antimicrobial adverse event during OPAT, *n* (%)	3 (15%)
Acute kidney injury	1 (5%)
Drug hypersensitivity rash	1 (5%)
* C. difficile* superinfection	1 (5%)
Unplanned readmission during OPAT, *n* (%)	6 (30%)
Congestive cardiac failure	2 (10%)
Fever for investigation	2 (10%)
Cardiac arrhythmia	1 (5%)
Antimicrobial adverse event	1 (5%)
Relapse of bacteraemia within 6 months of diagnosis, *n* (%)	1 (5%)
Mortality, *n* (%)	
Within 30 days of diagnosis	1 (5%)
Within 1 year of diagnosis^a^	3 (15%)

LVAD, left ventricular assistant device.

a One year follow-up data incomplete for 2 patients.

**Figure 1.**
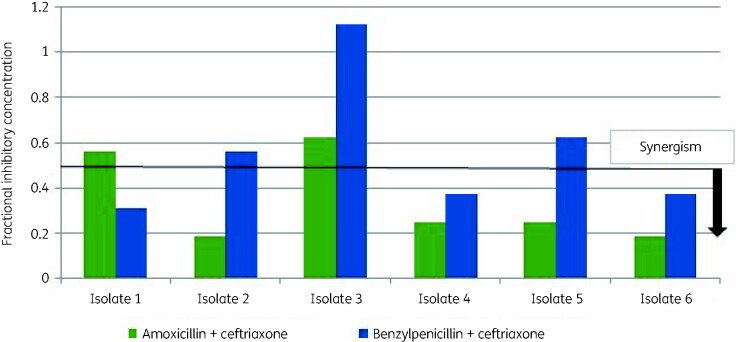
Comparison of fractional inhibitory concentrations for amoxicillin and ceftriaxone compared to benzylpenicillin and ceftriaxone amongst six *E. faecalis* isolates causing endovascular infection. A fractional inhibitory concentration index ≤0.5 indicates synergism.^12^

All enterococci were *E. faecalis*, and for the six isolates further characterized, the median benzylpenicillin MIC (1 mg/L, IQR 0.5–1 mg/L) was lower than the median amoxicillin MIC (4 mg/L, IQR 4–4 mg/L). Three isolates (50%) were high-level aminoglycoside resistant. Ceftriaxone MICs were all 256 mg/L. For all but one of the isolates, the combination of amoxicillin and ceftriaxone demonstrated a stronger synergistic relationship as shown by lower FICIs, with the criteria for synergy being met for 4 isolates (66%), versus 3 out of 6 (50%) for benzylpenicillin and ceftriaxone (Figure 1). The isolates belonged to ST179 (*n = *2), ST502 (*n = *2), ST56 (*n = *1) and ST6 (*n = *1).

## Discussion

As far as we are aware, this is the most comprehensive study of enterococcal endovascular infection management using benzylpenicillin and ceftriaxone in the outpatient setting, and based on our clinical observations and *in vitro* data caution should be exercised when utilizing this combination.

In contrast to the combination of amoxicillin and ceftriaxone, published outpatient experience with benzylpenicillin and ceftriaxone is limited to seven patients all of whom had favourable outcomes.^6^^,^^7^ In our cohort of older, comorbid patients, nearly half of whom had infections involving prosthetic material, patients had a higher rate of OPAT-related, unplanned readmissions (30%) than previously described,^6^^,^^7^ mostly due to relapsed fever or cardiac dysfunction. Published rates of hospital readmission from OPAT range from 3.5%–18%,^15^ however patients with enterococcal infections are known to have a threefold higher risk of adverse outcomes during OPAT, potentially explained by higher rates of illness acuity and use of multiple antimicrobials concurrently.^16^ The frequency of microbiologically proven relapse (5%) and one year mortality (15%) that we observed were comparable to the wider published experience using amoxicillin in combination with ceftriaxone, in which relapses occur in 0%–14% and one year mortality ranged from 17%–26%.^1^

Contrary to results of time–kill testing of a single *E. faecalis* isolate that failed to demonstrate synergy between benzylpenicillin and ceftriaxone,^6^ we did demonstrate synergism for this combination, albeit in only 3 of 6 (50%) isolates, with FICIs consistently inferior to the amoxicillin and ceftriaxone combination. Whilst amoxicillin and benzylpenicillin appear to exhibit similar capacity for synergy with aminoglycosides amongst enterococci,^17^ alterations in penicillin binding proteins may explain varying affinity for different β-lactams,^18^ and hence synergism between particular penicillins and cephalosporins. For example, when compared with other cephalosporins, ceftaroline exhibits a greater degree of synergy when part of a dual β-lactam combination.^18^ Our data suggests different penicillins have varying capacity for synergism with any given cephalosporin.

Given the paucity of clinical data and *in vitro* findings, outpatient alternatives to the combination of benzylpenicillin and ceftriaxone should be considered. For example, outpatient infusion of 2 g of amoxicillin every 6 h avoids the restrictions imposed by antimicrobial instability.^1^ However, antimicrobials delivered less frequently are better suited to OPAT, and data is emerging describing outpatient management of enterococcal endocarditis using teicoplanin or daptomycin once daily, or dalbavancin once weekly.^1^ Based on *in vitro* synergy testing results, daptomycin has also been successfully utilized in combination with ceftaroline via OPAT.^19^ In circumstances where clinicians would prefer to continue to use synergistic gentamicin, reducing the duration of gentamicin therapy may limit nephrotoxicity without compromising efficacy.^1^ Finally, amongst a selective patient population with endocarditis, of whom 24% had enterococcal infections, a recent randomized controlled trial demonstrated non-inferiority of predominantly oral outpatient therapy compared with inpatient parenteral therapy.^1^

A comprehensive assessment of clinical outcomes is prohibited by our small sample size, retrospective study design and the lack of a comparator group. Although hypothesis-generating only, our findings warrant further exploration as antimicrobial adjustment during transition to OPAT should not compromise patient outcomes. We acknowledge that correlation of *in vitro* synergy testing with clinical outcomes is limited,^12^ and variation in ceftriaxone dose frequency may have impacted OPAT outcomes, particularly as recent pharmacokinetic data suggests once daily dosing may be insufficient to achieve concentrations required for synergism with penicillins.^20^ Amoxicillin steady-state concentrations achieved during continuous infusions were recently described,^21^ and it has been suggested that non-bactericidal amoxicillin concentrations may move into the bactericidal range when co-administered with ceftriaxone.^4^ However, the relative impact on MBCs of ceftriaxone in combination with benzylpenicillin compared with amoxicillin is unknown. Finally, as antimicrobial synergy amongst enterococci is strain specific^5^ our observations would benefit from reproduction in a larger number of more geographically diverse isolates.

### Conclusions

On the basis of lower rates of synergistic antimicrobial interaction and a significant proportion of unplanned readmissions, clinicians should exercise caution when treating enterococcal endovascular infection using benzylpenicillin and ceftriaxone in the outpatient setting.
